# [^18^F]FMISO PET in metastatic neuroendocrine tumours: a pilot study

**DOI:** 10.22038/aojnmb.2025.83664.1611

**Published:** 2025

**Authors:** David L Chan, Alice Conner, Nick Pavlakis, Elizabeth Bailey, Alireza Aslani, Kathy Willowson, Connie Diakos, Elizabeth J Bernard, Stephen Clarke, Alexander Engel, Paul J Roach, Dale L Bailey

**Affiliations:** 1 Faculty of Medicine and Health, University of Sydney, Camperdown, Australia and Department of Medical Oncology, Royal North Shore Hospital, St Leonards, Australia; 2 Faculty of Medicine and Health, University of Sydney, Camperdown, Australia; 3 Department of Nuclear Medicine, Royal North Shore Hospital, St Leonards, Australia; 4 Faculty of Medicine and Health, University of Sydney, Camperdown, Australia

**Keywords:** Hypoxia, [^18^F]FMISO, [^68^Ga]DOTATATE, [^18^F]FDG, PET/CT, Neuroendocrine tumours (NETs)

## Abstract

**Objective(s):**

The phenomenon of peripheral [^68^Ga]DOTATATE avidity without central avidity (which we have termed a “DONUT") has been observed in neuroendocrine neoplasm (NEN) lesions. There has been speculation as to whether this is due to hypoxia, de-differentiated disease or other causes. The presence of hypoxia may have prognostic and therapeutic implications, and was evaluated in these lesions using the PET hypoxia imaging biomarker [^18^F]FMISO.

**Methods:**

Prospective pilot study in patients with metastatic NENs with at least one DONUT lesion (central [^68^Ga]DOTATATE non-avidity). [^18^F]FDG and [^18^F]FMISO scans were acquired within 60 days of the [^68^Ga]DOTATATE PET/CT. [^18^F]FMISO scans were acquired as a dynamic scan over 20 mins from injection with a delayed image at 2 hours. The dynamic acquisition was analysed quantitatively using a graphical approach yielding parametric images of Influx Rate Constant and Volume of Distribution. [^18^F]FMISO uptake within the identified DONUT hole on the 2 hr delayed scan was qualitatively scored by two experienced nuclear medicine physicians as: 0 (no uptake), 1 (uptake less than normal liver), 2 (uptake equal to normal liver), or 3 (uptake greater than normal liver).

**Results:**

Ten patients were enrolled with primary sites including pancreas (n=3), small bowel (n=3), rectum (n=2), duodenum (n=1) and lung (n=1). Six subjects were scored 1, three subjects were scored 2, and one subject was scored 3. All lesions evaluated were located in the liver. Quantitative [^18^F]FMISO parametric imaging showed evidence of increased uptake rate (Ki) in the photopenic areas of the DONUT lesions in 8/10 subjects. Surrounding uptake rate in normal liver was extremely low. In the qualitative delayed image assessment, only one subject demonstrated [^18^F]FMISO uptake greater than surrounding normal liver (small bowel primary, G2).

**Conclusion:**

Only one of ten patients with DONUT lesions demonstrated increased [^18^F]FMISO uptake rate on delayed static imaging. In contrast, dynamic imaging demonstrated increased [^18^F]FMISO uptake rate in the region of [^68^Ga]DOTATATE photopenia on 8 of 10 patients. Future research using [^18^F]FMISO in NEN patients should incorporate dynamic imaging.

## Introduction

 Neuroendocrine neoplasms (NENs) are an uncommon and heterogenous group of malignancies which are increasing in incidence and prevalence (1). NENs arise from neuroendocrine cells, most commonly in the gastrointestinal tract, lungs and pancreas. Over 20% of patients have metastatic disease at the time of diagnosis (2). NENs are highly diverse in clinical course, and treatment options are influenced by disease distribution and behaviour, as measured on biopsy and positron emission tomography/computed tomography (PET/CT) imaging. Whilst histological grade reflects the biological aggressiveness of the sampled tumour and predicts overall survival, this biomarker does not reflect behaviour in the entire extent of metastatic disease. Therefore, there is a pressing need for the development of new NEN biomarkers to guide optimal management.

 PET/CT imaging is increasingly utilised for whole-body imaging in solid tumours. It has the potential to map the underlying biology of each individual lesion, potentially yielding more information compared to biopsy which only evaluates one lesion. [^68^Ga]DOTATATE and [^18^F]FDG PET/CT scans are used extensively to image NENs. [^68^Ga]DOTATATE PET implies the overexpression of somatostatin receptors (SSTR) in well-differentiated, low grade NENs. 

 Accordingly, SSTR2 expression and [^68^Ga]DOTATATE uptake predict better prognosis in patients with metastatic NENs (3,4). [^18^F]FDG uptake reflects glycolytic activity, and correlates with tumour aggressiveness, higher grade, and reduced survival (5). The prognostic value of dual [^68^Ga]DOTATATE and [^18^F]FDG PET/CT (‘dual PET’) has been demonstrated by our group using the NETPET score, with the presence of [^18^F]FDG-avid /[^68^Ga]DOTATATE-non-avid lesions (score P5) associated with poor prognosis (6). Despite the considerable promise of dual PET in predicting NEN tumour aggressiveness, the proposed NETPET framework relies on each lesion having a relatively homogeneous uptake of radiopeptide. 

 A lesion which has partial [^18^F]FDG avidity and partial [^68^Ga]DOTATATE avidity in different areas would not be well classified by the current system, and the prognostic and therapeutic implications of this remain unclear.

**Figure 1:**
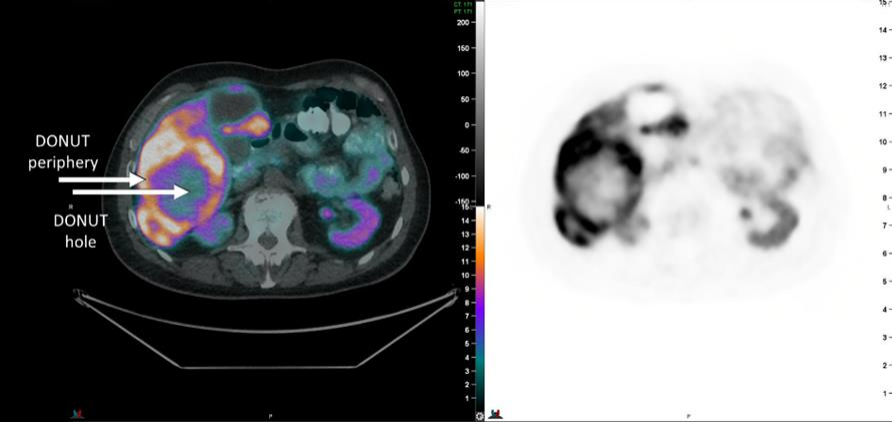
Example of a DONUT lesion identified on [^68^Ga]DOTATATE PET/CT. Both the non-avid centre (DONUT-hole) and the avid periphery must have measured >1cm each for inclusion

 The current study proposal follows on from a clinical observation that a minority of patients have a pattern of [^68^Ga]DOTATATE avidity in the periphery of a tumour lesion, with a non-avid centre, referred to here as a “DONUT” lesion “Figure 1”. The cause and significance of this pattern is unknown.

The lack of [^68^Ga]DOTATATE avidity may represent hypoxia, de-differentiation to more aggressive disease, or necrosis, each with their own clinical implications. Hypoxia is a common feature of solid tumours, and is associated with poor prognosis, metastasis, and resistance to therapy. Hypoxia-inducible factor-1α (HIF-1α) is a key regulator of the hypoxic response, and promotes angiogenesis, metabolism, and cell survival through downstream proteins (7). In patients with resected grade 1 and 2 ileal NENs, expression of downstream proteins CAIX and CXCR4 was correlated with higher grade, larger tumour size, the presence of lymph node metastasis, and lower survival (8). Similarly, high HIF-1α and low SSTR2 staining predict for shorter survival in resected gastroenteropancreatic NENs (GEPNENs), while low HIF-1α and high SSTR2 predict an indolent phenotype (9). 

 Hypoxia also confers chemo- and radio-resistance by limiting drug delivery to hypoxic regions and reducing the free radicals that make radiotherapy effective. Therefore, hypoxic tumours may require supratherapeutic doses of radiotherapy or treatment with combined chemoradiotherapy in order for such therapy to be effective.

 In contrast to hypoxia, de-differentiated/ poorly differentiated neuroendocrine carcinomas exhibit a loss of SSTR, and are highly proliferative and aggressive. Platinum based chemotherapy is typically used, however, median survival remains low (10). Necrosis can result from rapid tumour growth leaving the centre of the lesion without sufficient blood supply, and is an independent predictor of poor prognosis in resected pancreatic NENs (11). 

 Necrotic lesions may be detected by cross-sectional imaging alone, and in isolation are usually monitored. Therefore, elucidating the aetiology of the “DONUT” phenomenon can help clinicians optimise therapy for patients with NEN.

 In the current study, we aimed to use [^18^F]FMISO PET/CT to investigate the role of hypoxia in ten NEN patients with observed DONUT lesions and advanced disease. [^18^F]FMISO is a nitroimidazole compound which has a partition coefficient close to unity (12). [^18^F]FMISO PET/CT imaging is often used in head and neck cancer to image areas of hypoxia (13) as well as other cancers such as glioma (14). Despite this, there is a lack of data evaluating [^18^F]FMISO uptake in NENs. By comparing uptake on [^68^Ga]DOTATATE, [^18^F]FDG and [^18^F]FMISO PET/CT within the ‘DONUT-hole’ of [^68^Ga]DOTATATE non-avidity, using both static and dynamic acquisitions, we aimed to investigate the biological basis of this phenomenon that may have significant prognostic and therapeutic implications.

## Methods

### Subject cohort

 This prospective pilot study included ten adult patients with metastatic, histologically confirmed NEN, with a DONUT lesion on [^68^Ga]DOTATATE PET/CT as identified by an experienced nuclear medicine physician. For the purposes of this study, a DONUT lesion must have demonstrated [^68^Ga]DOTATATE avidity on the periphery of the lesion, with a non-avid centre DONUT-hole. Once identified, the DONUT lesion of interest was prospectively marked for later comparison with [^18^F]FMISO scans. Subjects must have also undergone an [^18^F]FDG PET as part of their normal clinical management within 60 days of the [^68^Ga]DOTATATE and [^18^F]FMISO PET/CT scans. Patients with a history of renal failure, those who required sedation for prior PET/CT imaging, or were unable to lie supine for the required period of time were excluded.

### Radiopharmaceutical preparation

 [^68^Ga]DOTATATE and [^18^F]FDG PET/CT scans were conducted as described in Chan 2017 (15). [^18^F]FMISO radiopharmaceutical preparation was synthesised in-house using cassette-based automated synthesis system (Scintomics GRP, Munich, GER). This method uses the NITTP (1-(2′-nitro-1′-imidazolyl)-2-O-tetrahydropyranyl-3-O-tosylpropanol) precursor and cartridge purification. The radiochemical purity (RCP) was analysed using Instant Thin Layer Chromatography (ITLC) and High Pressure Liquid Chromatography (HPLC). The mean RCP by ITLC was 99.54% (±0.10% SEM). The mean RCP by HPLC was 99.16% (±0.32% SEM). The mean decay-corrected final yield for the syntheses was 16.7% (±1.9% SEM), comparable to other studies (16-18).

### Acquisition of PET imaging and processing

 Images were acquired on a PET/CT scanner with extended axial FoV and time of flight (ToF) imaging capability (Siemens Biograph mCT/64, Knoxville, TN, USA). A 3-minute FMISO infusion (aiming for 375 MBq per patient) was commenced along with a 20-minute dynamic acquisition focussing on the index DONUT lesion, a one-field delayed static acquisition over the liver at 1 hour, and a 10-minute Whole Body acquisition 2 hours after injection from vertex to mid-thigh. This was in keeping with previous published studies of [^18^F]FMISO (19,20). Reconstruction parameters were 3D OSEM with two iterations and 21 subsets with PSF resolution recovery and ToF correction employed. Scatter correction was model-based and attenuation correction was CT-based. Post-reconstruction Gaussian filtering was performed with filter size of 5 mm.

### PET analysis

 The primary endpoint of this study was the quantification of [^18^F]FMISO uptake in the lesion of interest compared to background uptake. This uptake was scored qualitatively by a team of two experienced nuclear medicine physicians (PJR, EJB) as 0 (no uptake), 1 (uptake less than normal liver), 2 (uptake equal to normal liver), or 3 (uptake greater than normal liver). 

 Quantitative analysis was first carried out on static acquisition images. Exploratory image-based variables included SUV_mean_ for [^18^F]FDG and [^18^F]FMISO PET in the area of interest (corresponding to the [^68^Ga]DOTATATE DONUT-hole), and SUV_mean_ for the background normal liver using at least a 1cm spherical region of interest, resulting in calculation of tumour to background ratios (TBR) for each of the measures. Finally, Patlak-Rutland kinetic analysis of dynamic images from the first 20 mins after injection was performed which produced parametric maps for Ki (the rate constant for tissue uptake of FMISO) and Vd (volume of distribution) using in-house programmes previously described by our group (21). 

### Clinicopathological variables

 Clinicopathological variables (gender, age at diagnosis, age at [^18^F]FMISO PET/CT, primary site of disease, histological grade, functionality, and prior systemic therapies) were collected at study entry. Adverse events occurring within the 30 days subsequent to [^18^F]FMISO PET/CT administration were recorded and classified by Common Terminology Criteria for Adverse Events v5.0 (22), regardless of causation.

 Results for the degree of qualitative uptake were presented descriptively. Results for SUV, Ki and Vd were compared between DONUT lesions and uptake in the background liver using the Student’s t-test.

## Results

Ten subjects were successfully accrued between October 2018 and April 2021. All PET/CT imaging was conducted within a 75-day interval for each patient. The median administered dose of [^18^F]FMISO was 342 MBq (range, 157-397 MBq, 2-8 MBq/kg). Scans were successfully acquired in all subjects.

 The ten enrolled patients (n=7 male, median age 59 years) had a variety of primary tumour sites and grades. The most common primary sites were the small bowel and pancreas (n=3 each, Table 1). The median number of systemic therapies received prior to [^18^F]FMISO imaging was 1 (range 1-3). The median follow-up for surviving patients was 42.3 months, and four patients passed away in the 32 months following the last patient’s enrolment.

**Table 1:** Cohort summary characteristics

Characteristic	Subgroup	Subjects (n=10)	Subjects (%)
Gender	Male	7	70%
	Female	3	30%
Age	Mean age at diagnosis	59 (range 39-41)	100%
	Mean age at ^18^F-FMISO scan	62 (range 42-84)	100%
Primary site of disease	Small Bowel	3	30%
	Pancreas	3	30%
	Rectum	2	20%
	Duodenum	1	10%
	Lung	1	10%
Histological grade	Grade 1	2	20%
	Grade 2	6	60%
	Grade 3 (well-differentiated)	1	10%
	Bronchial (atypical carcinoid)	1	10%
Functional disease	Yes	3	30%
	No	7	70%
Lines of prior systemic treatments received (including somatostatin analogues)	One line	6	60%
	Two lines	3	30%
	Three lines	1	10%

***Static acquisition analysis***

 All ten lesions identified as DONUT lesions were located in the liver.

**Figure 2:**
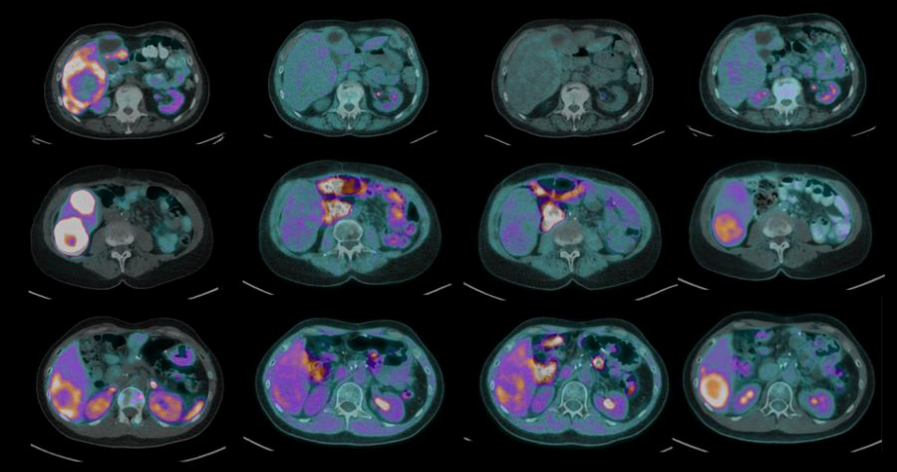
Representative examples of each score using fusion PET/CT. The region of interest on [^18^F]FMISO PET/CT corresponded to the [^68^Ga]DOTATATE PET/CT “DONUT-hole” of central non-avidity. Patients were scored based on [^18^F]FMISO uptake in this region compared to background: score 1 (uptake less than of normal liver), score 2 (uptake equal to normal liver), and score 3 (uptake greater than normal liver)

 One patient was scored 3, reflecting uptake in the DONUT lesion greater than liver background. Of the other nine subjects, three were scored 2 (uptake equal to liver background) and six were scored 1 (uptake less than liver background). Representative cross-sectional images centering on the DONUT-hole on patients with each score are presented in “Figure 2”. There were no discernible relationships between increased tumour grade or primary site and increased [^18^F]FMISO uptake.

 Exploratory variables relating to the SUV metrics of different scans as measured in the DONUT-hole are reported in Table 2. Of note, the tumour: background ratio on [^18^F]FMISO PET ranged from 0.5 to 1.3 on the 1 hour [^18^F]FMISO PET and 0.5 to 1.6 on the 2-hour image. In contrast, the tumour:background ratio for [^18^F]FDG PET ranged from 0.8 to 2.4.

**Table 2:** PET metrics in the DONUT-hole (central region of [^68^Ga]DOTATATE non-avidity)

**Patient ID**	**MISO score**	^18^ **F-FMISO 1h PET/CT**			^18^ **F-FMISO 2h PET/CT**			^18^ **F-FDG PET/CT**		
		**ROISUV**_mean_	**BackgroundSUV**_mean_	**TBR**	**ROISUV**_mean_	**BackgroundSUV**_mean_	**TBR**	**ROISUV**_mean_	**BackgroundSUVmean**	**TBR**
1	2	2.1	2.2	1.0	2.1	2.8	0.8	3.4	2.91	1.2
2	1	1.8	2.4	0.8	1.3	2.3	0.6	2.0	2.17	0.9
3	2	1.9	2.1	0.9	1.6	2	0.8	2.5	2.98	0.8
4	1	1.7	2.3	0.7	1.7	2.5	0.7	2.1	2.38	0.9
5	1	1.9	2.5	0.8	1.7	2.4	0.7	2.9	2.26	1.3
6	2	1.5	1.9	0.8	1.8	1.7	1.1	5.2	2.56	2.0
7	1	1.4	2.8	0.5	1.5	2.8	0.5	2.3	2.41	1.0
8	1	2.7	2.1	1.3	2.7	1.7	1.6	3.0	2.76	1.1
9	1	2.1	3.0	0.7	1.8	3.0	0.6	2.3	2.43	0.9
10	3	2.9	2.7	1.1	3.5	2.9	1.2	5.9	2.48	2.4

**TBR:** Tumour to Background Ratio

***Dynamic Image Analysis***

 On qualitative review of Ki and Vd maps, significantly increased Ki was observed to be present in 8 of the 10 “DONUT-hole” lesions, and significantly decreased Vd in 8 of the 10 lesions.

**Figure 3:**
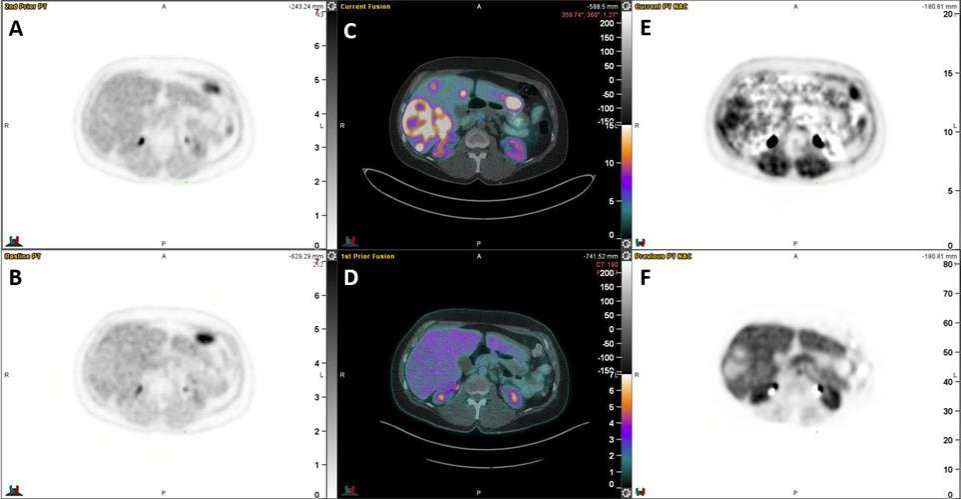
**A**) Static acquisition of liver 1h after FMISO injection. **B**) Static acquisition of liver 2h after FMISO injection. **C**) ^68^Ga-DOTA-TATE PET/CT fusion image, showing the “DONUT” lesion. **D**) ^18^F-FDG PET/CT fusion image. **E**) Ki map from Patlak-Rutland analysis of FMISO dynamic acquisition. **F**) Vd map from Patlak-Rutland analysis of FMISO dynamic acquisition

 The Ki mean was higher in the “DONUT-hole” lesions compared to background (mean 0.14%/

min vs 0.053%/min, mean difference 0.083%/min, 95% CI 0.023-0.143, p=0.01). The Vd was lower in the 10 lesions compared to background (mean 0.67 vs 1.20, mean difference -0.54, 95% CI -0.704 to -0.366, p<0.0001). Illustrative Ki and Vd images are provided in “Figure 3”.

***Safety***

 All ten patients completed study procedures as planned. Two of the ten patients reported adverse events of any grade (pain n=2, nausea n=1) from any cause within the 30 days following [^18^F]FMISO administration; no patients reported G3+ adverse events.

## Discussion

 It is known that tumour hypoxia is associated with cancer progression by the means of neovascularization, acquisition of the epithelial to mesenchymal transition phenotype, and development of metastases, whilst also conferring radioresistance (7, 23). 

 Furthermore, [^18^F]FMISO is known to be a powerful tool for studying hypoxia in vivo. 

 Studies in rectal and head and neck cancer show that hypoxia (as shown by [^18^F]FMISO PET) is a poor prognostic factor in this setting (24), and resolution of [^18^F]FMISO uptake after chemoradiation is associated with improved prognosis in gliomas (25).However, the biological role of hypoxia in patients with neuroendocrine tumours has not been well defined to date. We therefore planned the current study to investigate the potential role of hypoxia in NENs with its attendant prognostic

and therapeutic implications.

 To our knowledge, we describe here the first reported results of [^18^F]FMISO PET/CT imaging in patients with advanced NEN. Only one of the ten patients demonstrated significant [^18^F]FMISO uptake on static acquisition images. 

 Intriguingly, analysis of dynamic acquisition images showed that the majority of scans exhibited an increased rate of uptake (Ki) by Patlak-Rutland analysis and a decreased Vd in the “DONUT-hole”, but these results need to be interpreted with caution in a pilot study of 10 patients. Given the design of [^18^F]FMISO as an agent of diffusion (26), dynamic analysis may reflect the kinetics of perfusion, in that the lack of blood supply in the centre of a lesion may allow influx but not efflux of FMISO, potentially explaining the increase in Ki noted on dynamic imaging in this study. Indeed, contrasting findings between static and dynamic imaging have been noted in prior FMISO PET studies (27–29). If the role of [^18^F]FMISO imaging is confirmed in subsequent studies, radiation to hypoxic volumes or adding hypoxia-activated therapies may be considered in addition to standard systemic therapy as suggested in other tumour types (30).

 One of the strengths of this study is the prospective design and homogenization of imaging technique. We investigate the combination of [^68^Ga]DOTATATE PET, [^18^F]FDG PET and [^18^F]FMISO PET for the first time. The analysis of dynamic acquisitions provides a potential path to future [^18^F]FMISO PET/CT research in NENs and indeed other tumour types. We acknowledge the limitations of the current study including the small number of patients as well as the fact that two patients had a lower dose due to low yields in the manufacturing process. We also acknowledge that some more recent studies have analysed [^18^F]FMISO uptake via acquisitions at the 2 and/or 4 hour timepoints (14), as opposed to 1 and 2 hours in the current study, but also note that there is no established protocol thus far to determine the optimal scanning timepoints.

 The current study does not show a high incidence of [^18^F]FMISO uptake on delayed static imaging. Therefore, it argues against hypoxia playing a significant role in the milieu of metastatic NET lesions. Future studies of [^18^F]FMISO should include dynamic acquisitions given the intriguing findings we have demonstrated in the current study. Future avenues for research include the correlation of hypoxia markers in fresh tissue biopsies and [^18^F]FMISO PET avidity, investigation as to whether certain treatments (e.g., peptide receptor radionuclide therapy (PRRT) or chemotherapy) may induce hypoxia, and if a trigger for hypoxia is found (20) the effectiveness of different treatments for hypoxic NEN lesions. The recent suggestion on splicing analysis that CAIX is potentially associated with hypoxia in metastasis-like primary (MLP) subtypes of pancreatic NEN (31) may argue for novel CAIX-based radiotracers in pancreatic NEN patients with such findings.

## Conclusion

 [^18^F]FMISO uptake on delayed imaging is not commonly observed in NEN patients with DONUT lesions, arguing against a significant role of hypoxia in their pathogenesis. Increased [^18^F]FMISO uptake rate on dynamic imaging was observed, however, in 8 out of 10 subjects. 

 Further studies of FMISO PET incorporating dynamic acquisitions are warranted to further examine the role of hypoxia in NEN and its implications for therapy.

### List of Abbreviations

3D-OSEM: Third Dimensional Ordered Subset Expectation Maximization

CAIX: Carbonic Anhydrase 9

CT: Computed Tomography 

CXCR4: Chemokine Receptor type 4

FDG: Fluorodeoxy Glucose

FMISO: 18-Fluoromisonidazole 

FoV: Field of View

Ga: Gallium

GEPNEN: Gastroenteropancreatic neuroendocrine tumour

HIF-1α: Hipoxia-inducible factor-1α

HPLC: High Pressure Liquid Chromatography

ITLC: Instant Thin Layer Chromatography

Ki: Rate Constant of Tissue Uptake (%/min)

MBq: Megabecquerel

MLP: Metastasis-Like Primary 

NEN: Neuroendocrine Neoplasm

NET: Neuroendocrine Tumour 

PET: Positron Emission Tomography 

PSF: Point Spread Function 

RCP: Radiochemical Purity

SEM: Standard Error of the Mean 

SSTR: Somatostatin Receptor

SUV: Standardized Uptake Value

TBR: Tumour Background Ratio 

ToF: Time of Flight

Vd: Volume of distribution

## Data Availability

The datasets generated and/or analyzed during the current study are not publicly available due the risk of individual privacy breaches but are available from the corresponding author on reasonable request.
